# Overexpression of programmed cell death 5 in a mouse model of ovalbumin-induced allergic asthma

**DOI:** 10.1186/s12890-016-0317-y

**Published:** 2016-11-15

**Authors:** Xiaolin Diao, Juan Wang, Hong Zhu, Bei He

**Affiliations:** Department of Respiratory Medicine, Peking University Third Hospital, No. 49 Huayuan North Road, Haidian District Beijing, 100191 China

**Keywords:** Asthma, Programmed cell death protein 5, Lung function, Inflammation, Caspase-3

## Abstract

**Background:**

Programmed cell death 5 (PDCD5) was first identified as an apoptosis-promoting protein and involved in some autoimmune diseases and inflammatory processes. Our previous study demonstrated greater expression of serum PDCD5 in asthmatic patients than controls. This study aimed to further explore the significance of PDCD5 in mice with induced allergic asthma.

**Methods:**

We divided 16 female mice into 2 groups: control (*n* = 8) and allergen (ovalbumin, OVA)-challenged mice (*n* = 8). The modified ovalbumin inhalation method was used to generate the allergic asthma mouse model, and the impact of OVA was assessed by histology of lung tissue and morphometry. The number of cells in bronchoalveolar lavage fluid (BALF) was detected. Pulmonary function was measured by pressure sensors. PDCD5 and active caspase-3 levels were detected.

**Results:**

The expression of PDCD5 was higher with OVA challenge than for controls (*p* < 0.05). PDCD5 level was correlated with number of inflammatory cells in BALF and lung function. Moreover, active caspase-3 level was increased in the OVA-challenged mice (*p* < 0.001) and correlated with PDCD5 level (*p* = 0.000).

**Conclusions:**

These data demonstrate an association between level of PDCD5 and asthma severity and indicate that PDCD5 may play a role in allergic asthma.

**Electronic supplementary material:**

The online version of this article (doi:10.1186/s12890-016-0317-y) contains supplementary material, which is available to authorized users.

## Background

Allergic asthma is a chronic inflammatory disorder of the airways; many cells, such as lymphocytes, mast cells, eosinophils, smooth muscle cells, and cellular elements, contribute to its pathophysiological processes. The frequent occurrence of injury and repair initiated by chronic inflammation could lead to structural changes in the airway, collectively termed airway remodeling, which is characterized by epithelial injury, sub-epithelial fibrosis, enhanced deposition of extracellular matrix proteins, goblet cells and mucous gland hypertrophy and increased airway smooth muscle mass [1]. Airway inflammation and remodeling are two characteristics of asthma [2]. The development of chronic airway inflammation depends on the continuous recruitment of inflammatory cells and their subsequent activation. Duncan et al. demonstrated that patients with mild asthma had a significantly lower percentage of sputum eosinophils and a significantly higher eosinophil apoptotic ratio (AR) than those with moderate or chronic severe asthma [3]. Increasing evidence has shown that a dysregulation in programmed cell death mechanisms of both mobile and resident cells of the airways may directly contribute to the development of asthma as well as its clinical severity [3–6].

Programmed cell death or apoptosis is a form of cellular suicide widely observed in nature. Duncan et al. demonstrated a significant correlation of reduced apoptosis in eosinophils present in induced sputum with asthma severity [3]. Caspases are important regulators of apoptosis. Caspase-3 is considered the key executioner caspase in apoptosis [7–9]. The level of apoptotic caspase-3 protein was found increased in bronchial epithelial cells or lung tissues in asthma [10–12]. In contrast, its level was markedly decreased in pulmonary vascular smooth muscle of an asthma model [13]. Apoptosis is also a central and essential process in the resolution of inflammation. The resolution of eosinophilic inflammation of asthma relies on corticosteroids. Steroids can induce apoptosis of lung eosinophils and enhance the recognition and engulfment of apoptotic eosinophils by macrophages or bronchial epithelial cells [14]. However, the regulation mechanism of apoptosis in asthma control is not clear.

Programmed cell death 5 (PDCD5) was first identified as an apoptosis-promoting protein [15]. It was associated with DNA damage-induced apoptosis by interacting with the histone acetyltransferase Tip60 and was phosphorylated in vitro or in vivo by the multifunctional kinase CK2 [16, 17]. Clinically, PDCD5 is involved in some autoimmune diseases and inflammatory processes such as lupus nephritis [18], rheumatoid arthritis [19], osteoarthritis [20], hepatitis [21] and sepsis [22]. However, few studies have focused on whether and how PDCD5 is involved in asthma. We previously found serum PDCD5 level higher in patients with asthma than controls and negatively correlated with several indexes of lung function [23].

Here we established a mouse model of allergic asthma and further explored the significance of PDCD5 in bronchial asthma.

## Methods

### Reagents

Chicken egg ovalbumin (OVA) and aluminium hydroxide powder were from Sigma-Aldrich (St Louis, MO, USA). Periodic acid-Schiff staining (PAS) and Masson’s trichrome staining (Masson) kits were from Shanghai Yuanye Bio-Technology. Mouse anti-PDCD5 monoclonal antibody and the PDCD5 ELISA kit were gifts from Prof. Yingyu Chen (Center for Human Disease Genomics, Peking University, Beijing). Anti-active caspase 3 antibody was from Abcam (Cambridge, MA, USA).

### Animals

This study was carried out in strict accordance with the recommendations in the Guide for the Care and Use of Laboratory Animals of the US National Institutes of Health. The protocol was approved by the Committee on the Ethics of Animal Experiments of Peking University Health Science Center (Permit No.: LA2011-062). All surgery was performed with animals under urethane anesthesia, and all efforts were made to minimize suffering.

BALB/c mice (female, 6–8 weeks old) were obtained from the Department of Laboratory Animal Science (Peking University Health Science Center, Beijing). They were kept under pathogen-free conditions and had free access to food and water during experiments. Mice were randomly divided into 2 groups (*n* = 8 each): control and allergen (OVA)-challenged group.

### Establishing allergic asthma mouse model

The modified OVA inhalation method was used to generate the allergic asthma mouse model as described [24]. Briefly, the protocol consisted of an intraperitoneal injection of 20 μg OVA and 2.25 mg aluminum hydroxide gel on day 1 and 14. On day 21, the mice were placed in a plexiglass chamber (40 × 30 × 15 cm) connected to an ultrasonic nebulizer (model YC-Y800, Yadu, Beijing) and subjected to repeated bronchial allergen inhalation with 30 ml OVA (2.5% weight/volume diluted in sterile physiological saline) for 30 min/day on 3 consecutive days/week for up to 8 weeks. Control animals received only saline. Mice were killed 24 h after the last exposure.

### Respiratory function measurement

The mice were anesthetized with 10% urethane injected intraperitoneally and were intubated endotracheally by use of a trocar. Respiratory function was detected by using an animal ventilator (AD Instruments, Australia) connected to a pressure sensor. A “Y”-type trachea tube connected to flow sensors and pressure sensors was inserted into the trachea. A ventilator was connected to a flow sensor. The tidal volume was 10 mL/kg and the respiratory rate was 60 times/min. The Powerlab multi-lead physical instrument was connected to flow and pressure sensors. The peak inspiratory flow (PIF), peak expiratory flow (PEF), intra-airway pressure (IP) and maximum rising slope of IP (IP slope) were measured, and data were analyzed by using Chart 4.1 (AD Instruments, Australia).

### Bronchoalveolar lavage fluid (BALF) cytology

Mouse lungs were sequentially lavaged three times with 0.5 ml physiological saline. Recovered aliquots of BALF were pooled. BALF cells were pelleted by centrifugation at 2000 rpm for 5 min. Cell differentials were determined with cytospin preparations stained with Wright–Giemsa, and 200 cells were counted. The supernatant was stored at −80 °C.

### Analysis of lung histopathology

After BALF cytology, lungs were inflated with 10% formalin and immersed in 10% formalin fixation solution. Paraffin-embedded lung sections were stained with hematoxylin and eosin (H&E) for inflammatory cell infiltration and proliferation of smooth muscle, periodic acid-Schiff (PAS) for goblet cells and Masson trichrome for airway fibrosis and collagen deposition. An expert respiratory pathologist blinded to treatment groups graded the extent of inflammation in the lungs according to a semi-quantitative scoring system Scores for inflammatory cell infiltration were 0, no inflammatory infiltrations; 1, sporadic inflammatory cells; 2, more maldistributed inflammatory cells, not gathered into groups; 3, a large amount of inflammatory cells, uniformly distributed but few gathered into groups; and 4, large amount of inflammatory cells gathered into groups.

### ELISA

The supernatant of BALF (1:10 diluted) was added to 96-well ELISA plates (100 μl/well) and incubated for 60 min at 37 °C. After washing, 1 μg/ml anti-PDCD5 antibody (100 μl/well) was added for incubation at 37 °C for 60 min. After multiple washes, TMB solution (100 μl/well) was added for incubation in the dark at room temperature for 15 min. Color development was stopped by adding 2 M H_2_SO_4_ (50 μl) and absorbance was measured at OD 450 nm (OD_450_).

### Immunohistochemistry (IHC)

Sections of 4 μm lung tissue were incubated with antibodies for PDCD5 (1:300) and active caspase-3 (1:100) at 4 °C overnight. IHC staining involved use of DAB (DAKO, Carpinteria, CA, USA). The signal was recorded in four grades by intensity of staining: 0, 1+, 2+ and 3+. The percentages of PDCD5-positive cells or active caspase-3 -positive cells were also recorded in four categories: 1 (0–25%), 2 (26–50%), 3 (51–75%) and 4 (76–100%). The sum of the intensity and percentage scores was used as the final staining score.

### Statistical analysis

Data are presented as mean ± SD. One-way ANOVA was used to compare multiple samples and Student’s independent *t* test to compare two groups. Pearson correlation coefficient (r) was calculated to assess correlations between nonparametric and parametric data. *P* < 0.05 was considered statistically significant. Data were analyzed by using SPSS 13.0 and GraphPad Prism 5.0.

## Results

### Respiratory function was markedly changed in OVA-challenged mice

**Fig. 1:**
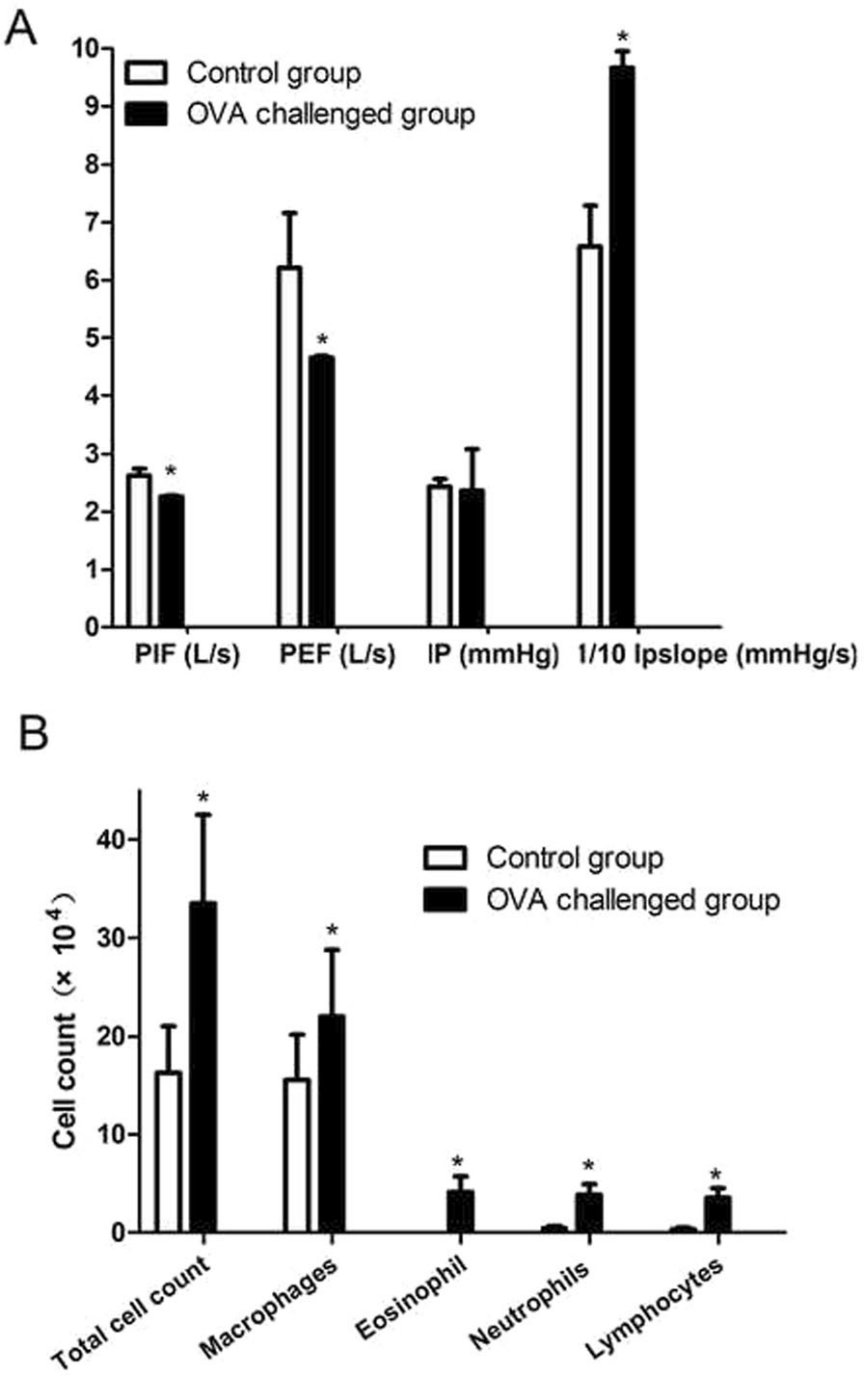
Clinicopathological indexes in mouse groups: control (*n* = 8) and allergen (ovalbumin, OVA)-challenged mice (*n* = 8). **a** Lung function. **b** Counts of inflammatory cells in bronchoalveolar fluid (BALF). Data are mean ± SD. ^*^ *P* < 0.05 compared with control. PIF, peak inspiratory flow; PEF, peak expiratory flow; IP, intra-airway pressure; IP slope, maximum rising slope of IP

Compared with controls, OVA-challenged mice showed lower PIF and PEF (2.26 ± 0.02 vs 2.62 ± 0.12 L/s, 4.65 ± 0.04 vs 6.21 ± 0.95 L/s, respectively, *p* < 0.05) (Fig. 1a). The IP slope was higher in OVA-challenged mice than controls (96.66 ± 2.88 vs 65.81 ± 7.07 mmHg/s, *p* < 0.05).

### Airway inflammation and airway remodeling after OVA sensitization

The total number of inflammatory cells in BALF was higher in OVA-challenged mice than controls (33.47 ± 9.07 vs 16.29 ± 4.72 × 10^4^, *p* < 0.05). Moreover, the counts of macrophages, eosinophils, neutrophils and lymphocytes were higher (*p* < 0.05) (Table 1, Fig. 1b).

**Table 1:** Inflammatory cell profile in bronchoalveolar fluid (BALF) in mice with and without ovalbumin (OVA) challenge

Group		Cell type count			
	Total cells (×10^4^)	Macrophages (×10^4^)	Eosinophils count (×10^4^)	Neutrophils (×10^4^)	Lymphocytes (×10^4^)
Control (*n* = 8)	16.29 ± 4.72	15.54 ± 4.65	0	0.45 ± 0.22	0.31 ± 0.19
OVA challenge (*n* = 8)	33.47 ± 9.07^*^	21.99 ± 6.75^*^	4.09 ± 1.61^*^	3.85 ± 1.07^*^	3.54 ± 1.00^*^

Data are mean ± SD

^*^ *P* < 0.05 compared with control

**Fig. 2:**
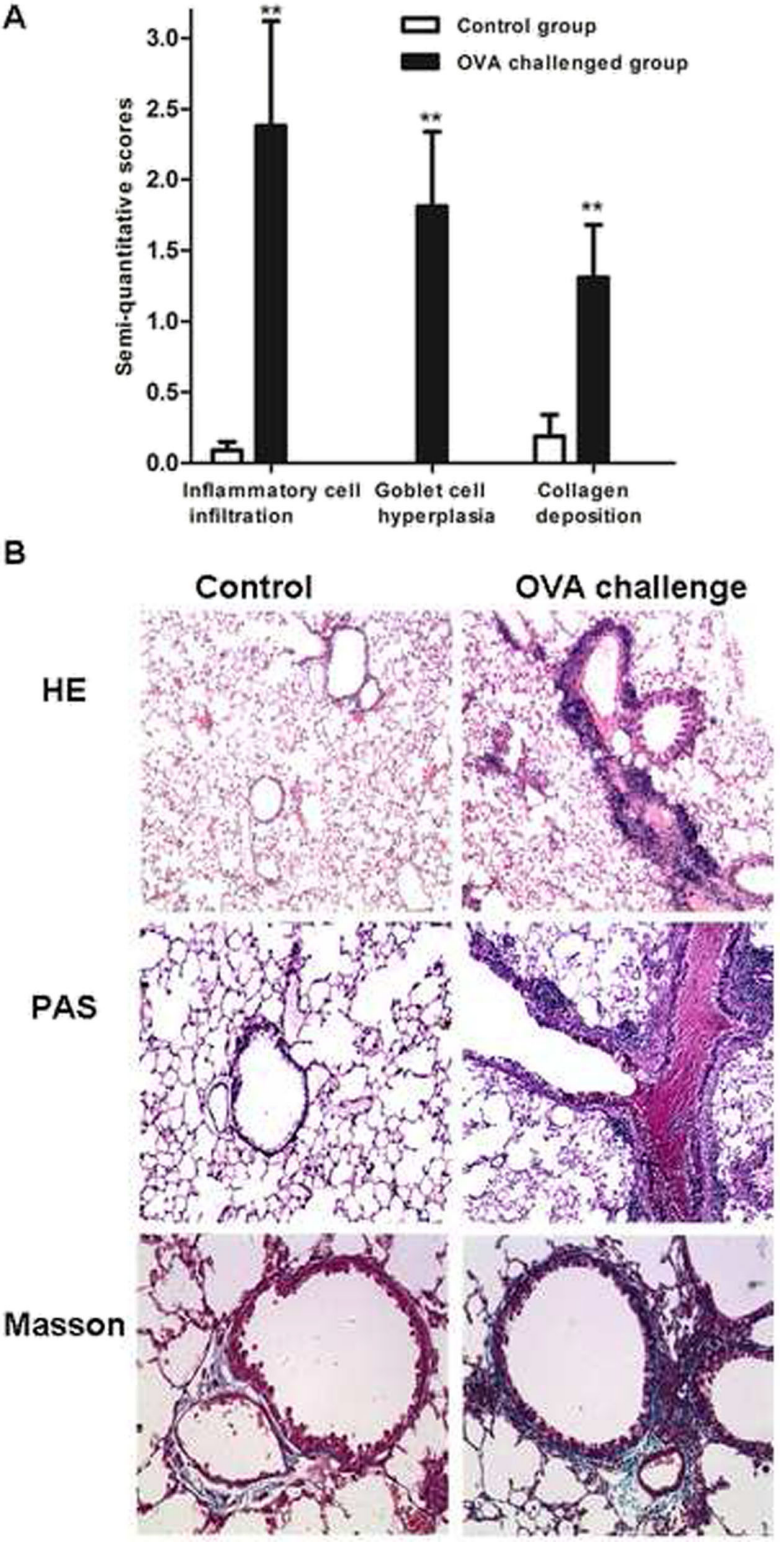
Comparison of pathological changes in control and OVA-challenged mice. **a** Scores of pathological indices. **b** Representative lung sections showing inflammatory cell infiltration and proliferation of smooth muscle cells with hematoxylin and eosin (HE) staining, goblet cell hyperplasia with periodic acid-Schiff (PAS) staining, and airway fibrosis and collagen deposition around airway with Masson trichrome staining. Data are mean ± SD. ^**^ *P* < 0.01 compared with control

The allergic asthma model showed increased inflammatory cell infiltration in airways and pulmonary vasculature, goblet-cell hyperplasia, smooth muscle cell proliferation, peribronchial fibrosis and collagen level as compared with controls (*p* < 0.01; Table 2, Fig. 2).

**Table 2:** Semi-quantitative scores for pathological indexes

Group	Inflammatory cell infiltration	Goblet-cell hyperplasia	Collagen deposition
Control (*n* = 8)	0.09 ± 0.06	0.00 ± 0.00	0.19 ± 0.15
OVA challenge (*n* = 8)	2.38 ± 0.74^**^	1.81 ± 0.53^**^	1.31 ± 0.37^**^

Data are mean ± SD

^**^ *P* < 0.01 compared with control

### Elevated PDCD5 level in BALF and lung tissue after OVA challenge

**Fig. 3:**
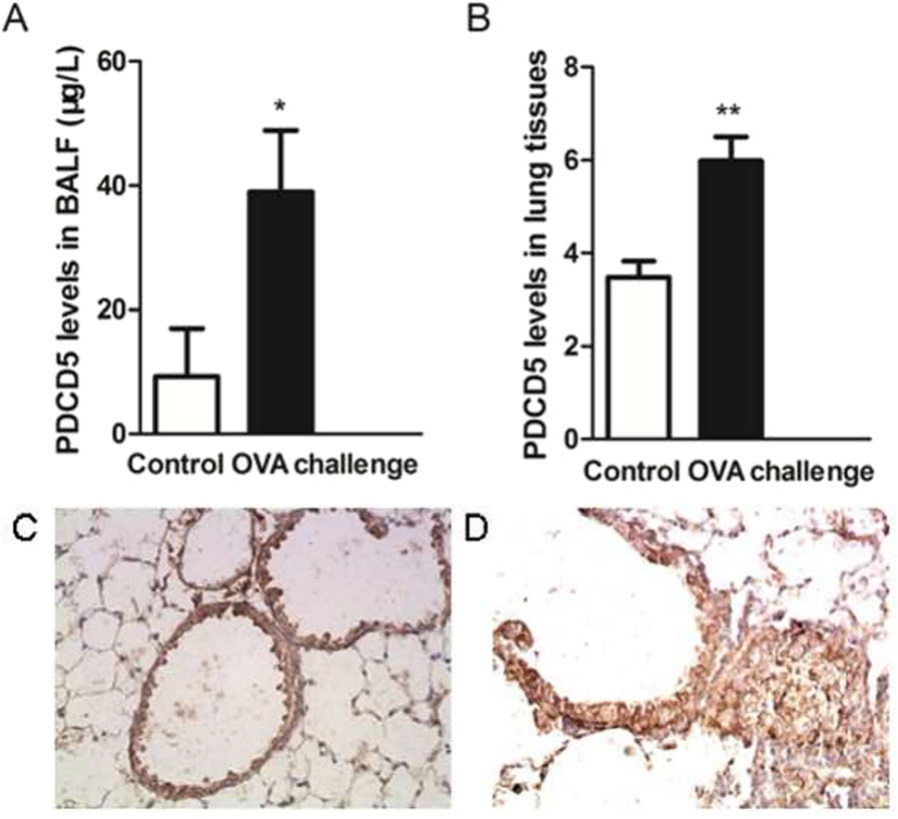
PDCD5 protein expression. **a** ELISA of PDCD5 protein level in BALF. **b** IHC of PDCD5 protein content in lung tissue. Representative PDCD5 IHC staining in lung sections of control (**c**) and asthmatic mice (**d**). Data are mean ± SD (*n* = 16). ^*^ *P* < 0.05 compared with control, ^**^ *P* < 0.01 compared with control

PDCD5 expression was increased in BALF of OVA-challenged mice and its protein level in BALF was higher (38.96 ± 9.96 vs 9.25 ± 7.76 μg/L, *p* < 0.05, Table 3). On IHC, PDCD5 protein content was increased in airway epithelial cells and inflammatory cells around airways (Fig. 3). PDCD5 protein staining was greater in OVA-challenged mice than controls (5.99 ± 0.52 vs 3.48 ± 0.35, *p* < 0.01) (Table 3).

**Table 3:** Programmed cell death 5 (PDCD5) level in BALF and lung tissue with and without OVA challenge

PDCD5	Control (*n* = 8)	OVA challenge (*n* = 8)
BALF (μg/L)	9.25 ± 7.76	38.96 ± 9.96^*^
Lung tissue	3.48 ± 0.35	5.99 ± 0.52^**^

Data are mean ± SD

^*^ *P* < 0.05 compared with control, ^**^ *P* < 0.01 compared with control

### Correlation between PDCD5 expression and clinicopathologic indexes

**Fig. 4:**
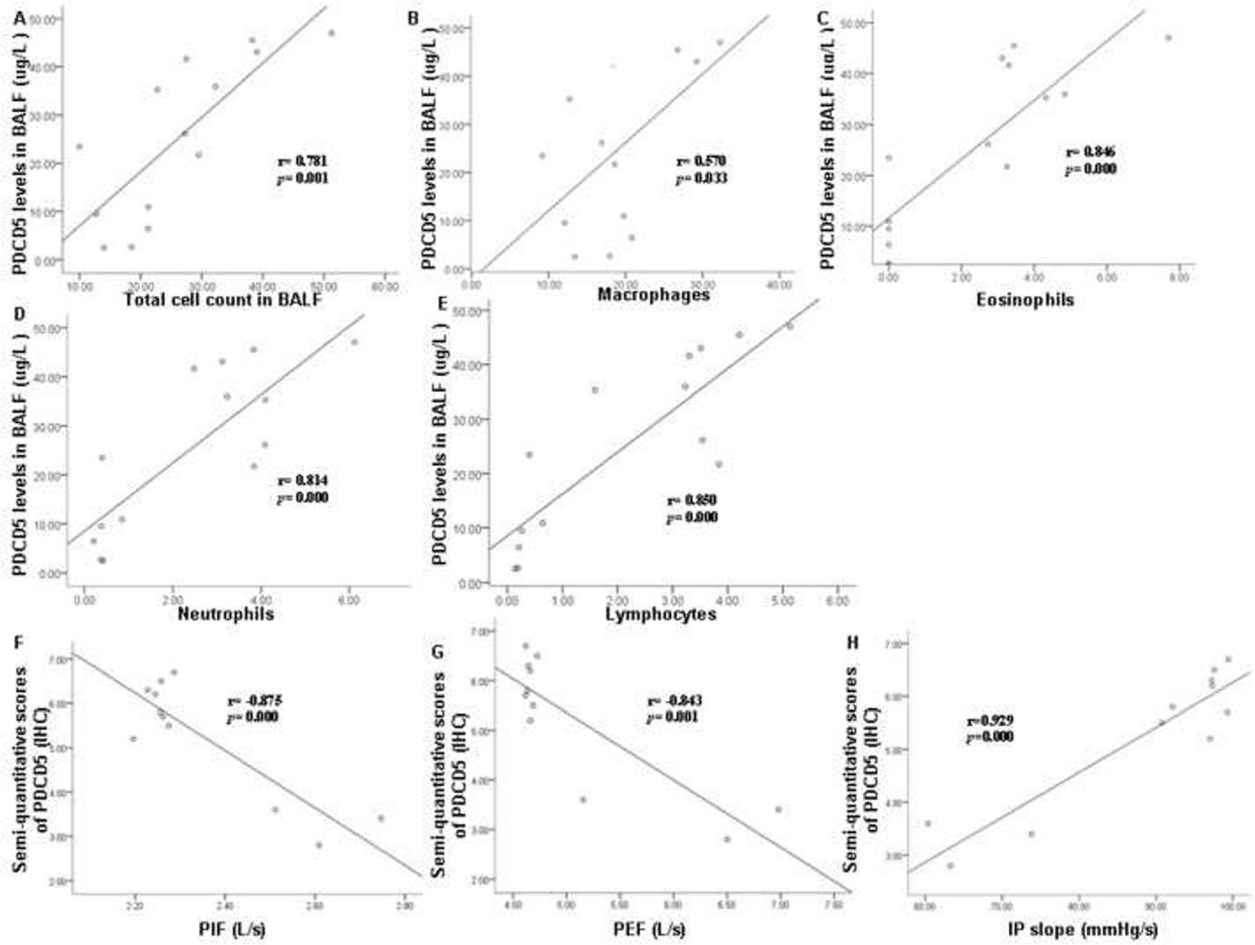
Correlation between PDCD5 expression and clinicopathologic characteristics. **a**-**e** Correlation between PDCD5 level and number of inflammatory cells in BALF, (**f**-**h**) Correlation between PDCD5 level in lung tissues and lung function

In BALF, PDCD5 level was positively correlated with total number of cells collected (*r* = 0.781, *p* = 0.001, *n* = 16) and total number of macrophages (*r* = 0.570, *p* = 0.033, *n* = 16), eosinophils (*r* = 0.846, *p* = 0.000, *n* = 16), neutrophils (*r* = 0.814, *p* = 0.000, *n* = 16) and lymphocytes (*r* = 0.850, *p* = 0.000, *n* = 16). In lung tissue, PDCD5 staining intensity was negatively correlated with PIF (*r* = −0.875, *p =* 0.000, *n* = 16) and PEF (*r* = −0.843, *p =* 0.001, *n* = 16) but positively with IP slope (*r* = 0.929, *p =* 0.000, *n* = 16) (Fig. 4). A similar correlation was found when analyzing PDCD5 level in BALF with lung function (Additional file 1: Figure S1).

### Correlation between active caspase-3 level and clinicopathologic indexes

**Fig. 5:**
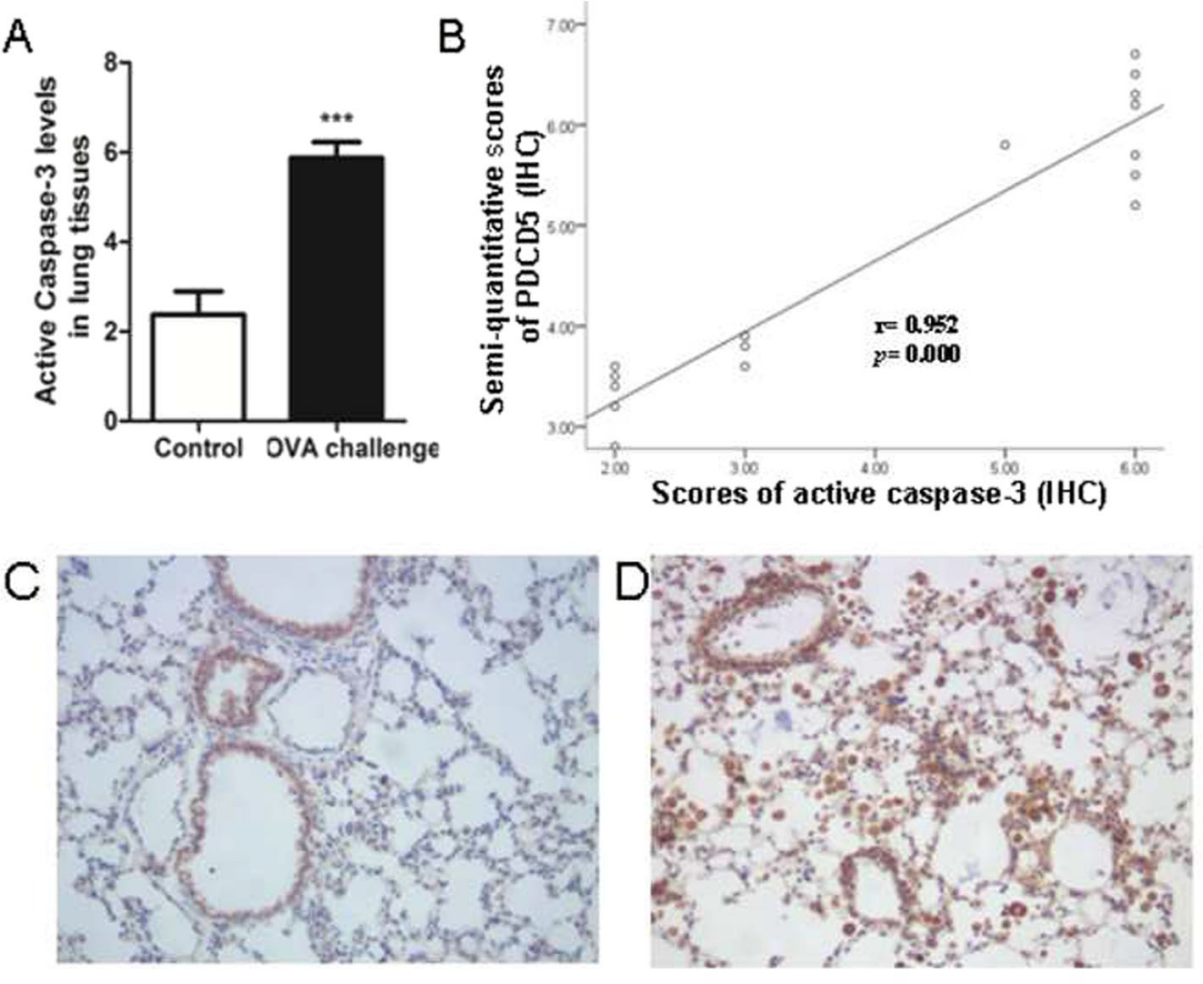
Active caspase-3 protein expression. **a** IHC of active caspase-3 protein content in lung tissue, (**b**) Correlation between PDCD5 and active caspase-3 level. Representative active caspase-3 IHC staining of lung sections of control (**c**) and OVA-challenged mice (**d**). Data are mean ± SD (*n* = 16). ^***^ *p* < 0.001 compared with control

Active caspase-3 showed positive staining in airway epithelial cells of bronchioles and bronchium of OVA-challenged mice. Its protein was expressed mainly in macrophages and some B lymphocytes and plasmocytes. Active caspase-3 level was higher in OVA-challenged mice than controls (5.875 ± 0.354 vs. 2.375 ± 0.518, *p* < 0.001). Moreover, PDCD5 and active caspase-3 levels were positively correlated (*r* = 0.952, *p* = 0.000, *n* = 16) (Fig. 5).

## Discussion

Our previous study demonstrated increased serum PDCD5 level in asthmatic patients, which was correlated with clinical manifestations and lung function [23]. In the present study, we successfully established a mouse model of allergic asthma. This is the first report to investigate the upregulation of PDCD5 in BALF and lung tissue of asthmatic mice and to demonstrate that such upregulation was correlated with number of inflammatory cells in BALF and lung function as well as active caspase-3 level. Moreover, activated caspase-3 level was increased and correlated with PDCD5 level in asthma. These data support our previous clinical findings and indicate that PDCD5 may play a role in allergic asthma and its level may be associated with asthma severity by regulating apoptosis.

The asthmatic mouse model is commonly used to study human asthma because of the similarities. OVA-challenged mice show pathological and clinical features similar to that observed in human allergic asthma. The total number and proportion of eosinophils in BALF was increased in our asthmatic mice. Lung function, especially PEF, was decreased; as well, we found inflammatory cell infiltration, goblet cell hyperplasia, increased mucus secretion and collagen deposition around airways.

PDCD5 was first characterized as upregulated in cells undergoing apoptosis [15]. Later its expression was found downregulated in various tumors [25–28] but upregulated in autoimmune diseases. PDCD5 was found involved in some autoimmune diseases and inflammatory processes, such as rheumatoid arthritis and psoriasis [29–31]. PDCD5 transgenic mice exhibited a systemic anti-inflammatory condition in autoimmune encephalomyelitis mice [32]. Later the authors found the anti-inflammatory effects of recombinant human PDCD5 (rhPDCD5) in a rat collagen-induced arthritis model and provided the comprehensive assessment of immunosuppressive pathways of rhPDCD [33]. However, few studies have investigated the role of PDCD5 in asthma. In the present study, our results imply a correlation between asthma and apoptosis and confirm that PDCD5 participates in airway inflammation and airway remodeling of asthma; downregulated expression of PDCD5 could reflect the relief of airway inflammation and remodeling.

Asthma in humans is due to not just allergy. Our previous study of PDCD5 in asthma patients did not specify whether asthmatic patients were allergic or not, whereas the mouse model in this study is an allergic asthma model. Considering the consistent correlation between PDCD5 expression and severity of asthma from clinical research to animal models, although we cannot regard PDCD5 as a potential biomarker for monitoring and controlling asthmatic severity, PDCD5 may play a role in allergic asthma.

Moreover, we found increased PDCD5 protein level in airway epithelium and inflammatory cells around airways. PDCD5 could induce apoptosis of many kinds of cells [34, 35]. Thus, with increased number of cells involved in inflammation and remodeling, more PDCD5 was produced to inhibit the progression of inflammation and remodeling. Accordingly, with reduced number of cells involved in inflammation and remodeling, PDCD5 expression was reduced. However, further studies of the underlying molecular mechanisms are required.

Caspases are a family of cysteine-dependent aspartate-directed proteases that play essential roles in apoptosis, necrosis, and inflammation [36]. There are two types of apoptotic caspases: initiators (apical) and effectors (executioner) [37]. Active effector caspases trigger the apoptotic process [7]. Caspase-3 has long been recognized as the key executioner caspase in apoptosis, and active caspase-3 is an early marker of apoptosis [7–9]. To further identify the correlation between asthma and apoptosis, we measured the level of active caspase-3. In agreement with previous studies, active caspase-3 protein was expressed mainly in epithelial cells and macrophages. In accordance with the increased expression of PDCD5 in OVA-challenged mice, active caspase-3 level was also increased in lung tissues of these mice. Several studies have reported that PDCD5 can promote the activation of caspase-3 [38–40]. Upregulated active caspase-3 indicates increased cell apoptosis during the development of asthma. Macrophages or bronchial epithelial cells can recognize and remove apoptotic eosinophils, so increased apoptotic epithelial cells or macrophages may lead to airway remodeling and accumulated eosinophils.

Zn is an important regulator of caspase-3 [41]. Truong-Tran et al. found adverse effects of Zn deficiency on the respiratory epithelium and a role for altered Zn homeostasis and caspase upregulation in asthma [42]. However, further study of the possible effect of a Zn-limited diet on caspase upregulation in asthmatic mice is needed.

## Conclusions

We found a correlation between PDCD5 expression and severity of asthma in OVA-induced asthmatic mice. PDCD5 may play a role in allergic asthma. Future studies are required to determine the underlying mechanism.

## Additional file

Additional file 1: Fig. S1. Correlation between PDCD5 level in BALF and lung function. (TIF 40 kb)

## Abbreviations

BALF: Bronchoalveolar lavage fluid
H&E: Hematoxylin and eosin
IHC: Immunohistochemistry
IP slope: Maximum rising slope of IP
IP: Intra-airway pressure
Masson: Masson’s trichrome staining
OVA: Ovalbumin
PAS: Periodic acid-Schiff staining
PDCD5: Programmed cell death 5
PEF: Peak expiratory flow
PIF: Peak inspiratory flow
SD: Standard deviation
