# Supplementary figures and images for: Overexpression of programmed cell death 5 in a mouse model of ovalbumin-induced allergic asthma
Source: BMC Pulm Med. 2016 Nov 15;16:149. doi: 10.1186/s12890-016-0317-y (PMC5109699; doi:10.1186/s12890-016-0317-y)

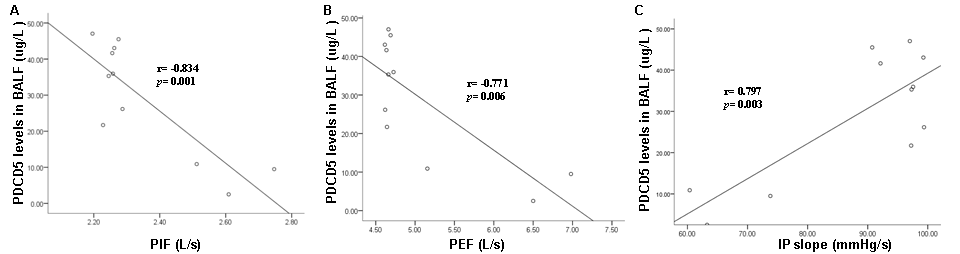

Supplement: Additional file 1: Fig. S1. — Correlation between PDCD5 level in BALF and lung function. (TIF 40 kb) [file 12890_2016_317_MOESM1_ESM.tif]
